# Supplementary material for: Acceptance and compliance with micronutrient powder (MNP) among children aged 6–23 months in northern Nigeria
Source: PLOS Glob Public Health. 2022 Oct 17;2(10):e0000961. doi: 10.1371/journal.pgph.0000961 (PMC10022258; doi:10.1371/journal.pgph.0000961)
Supplement: S1 Dataset — (ZIP) [file pgph.0000961.s008.zip › S8_Dataset/Spot Checks.zip!/Spot Checks/110715_K_DW_AD_2_BO.c.pdf]

SPOT CHECK RECORDING FORM

State: Kebbi LGA: D/wasagu Community: D/Daji

Sex of index child: F   Age of index child (in months): 20months

Spot Check Information

1. **Date of MNP distribution to household** (circle option below based on LGA name above)**:**
   - **Kebbi:** BirninKebbi&Jega LGA: **June 16**
   - **Kebbi:** Danko-wasagu: **June 17**
   - **Adamawa:**Song, Ganye: **June 18**
   - **Adamawa:**Guyuk **June 19**
2. **Date of Spot Check Observation: 11/07/15**
3. **Number of sachets remaining**(Ask the caregiver to show you and count the specific number of sachets remaining)**:**

   40

4. **Number of sachets that should remainusing one MNP sachet per day** (Analyst only)**:**

   37

5. **Number of sachets remaining / number of sachets should be remaining**(Analyst only)**:**

   ____40_____ / _____37____ = ____108.1_____ %

Probes

Question 1. You have **XX** MNP sachets remaining. Explain how easy or difficult it was to use the MNP product on a daily basis for your child 6 – 23 months? (Circle one answer)

Very Difficult **Difficult** **Easy** **Very Easy**

Question 2. Describe any specific challenges that you faced that made the MNP difficult to use on a daily basis?

It was very easy to use

Question 3. Describe specific aspects of the MNP product that you or your child enjoyed that made it easier to use on a daily basis?

I like it because it has increased her appetite a lot now and now she eats more. She doesn't normally eats well but now she does

Question 4. What questions do you have about the MNP based on your experience using it?

I don't have any problem using this product because I understand how to use the product and it wasn't a problem for me

Question 5. What specific recommendations do you have that we can do as a program to make the MNP easier to use on a daily basis?

You should bring more of the product to us since it is very easy to use and also very effecive
